# Antimalarial Drugs at the Intersection of SARS-CoV-2 and Rheumatic Diseases: What Are the Potential Opportunities?

**DOI:** 10.3390/medicina60071171

**Published:** 2024-07-19

**Authors:** Saule Abisheva, Kristina Rutskaya-Moroshan, Gulnaz Nuranova, Tansholpan Batyrkhan, Anilim Abisheva

**Affiliations:** 1 Department of Family Medicine №1, NJSC “Astana Medical University”, Astana 010000, Kazakhstan; saule_tabisheva@mail.ru (S.A.); tansholpan.batyrkhan@gmail.com (T.B.); aanelim@bk.ru (A.A.); 2 Department of Children’s Diseases with Courses in Pulmonology and Nephrology, NJSC “Astana Medical University”, Astana 010000, Kazakhstan; nuranova_21@mail.ru

**Keywords:** COVID-19, rheumatic diseases, antimalarial drugs, hydroxychloroquine

## Abstract

*Background and Objectives*: The coronavirus disease of 2019 (COVID-19) pandemic has posed a serious threat to humanity and is considered a global health emergency. Antimalarial drugs (ADs) have been used in the treatment of immuno-inflammatory arthritis (IIA) and coronavirus infection (COVID-19). The aim of this review is to analyze the current knowledge about the immunomodulatory and antiviral mechanisms of action, characteristics of use, and side effects of antimalarial drugs. *Material and Methods*: A literature search was carried out using PubMed, MEDLINE, SCOPUS, and Google Scholar databases. The inclusion criteria were the results of randomized and cohort studies, meta-analyses, systematic reviews, and original full-text manuscripts in the English language containing statistically confirmed conclusions. The exclusion criteria were summary reports, newspaper articles, and personal messages. Qualitative methods were used for theoretical knowledge on antimalarial drug usage in AIRDs and SARS-CoV-2 such as a summarization of the literature and a comparison of the treatment methods. *Results*: The ADs were considered a “candidate” for the therapy of a new coronavirus infection due to mechanisms of antiviral activity, such as interactions with endocytic pathways, the prevention of glycosylation of the ACE2 receptors, blocking sialic acid receptors, and reducing the manifestations of cytokine storms. The majority of clinical trials suggest no role of antimalarial drugs in COVID-19 treatment or prevention. These circumstances do not allow for their use in the treatment and prevention of COVID-19. *Conclusions*: The mechanisms of hydroxychloroquine are related to potential cardiotoxic manifestations and demonstrate potential adverse effects when used for COVID-19. Furthermore, the need for high doses in the treatment of viral infections increases the likelihood of gastrointestinal side effects, the prolongation of QT, and retinopathy. Large randomized clinical trials (RCTs) have refuted the fact that there is a positive effect on the course and results of COVID-19.

## 1. Introduction

The first severe acute respiratory syndrome coronavirus 2 (SARS-CoV-2) outbreak was registered in the Wuhan Province, China in 2019. The large-scale spread of infections led to the development of a global pandemic that poses a threat to humanity as a whole. In most cases, despite mild infection, about 5 to 15 percent of patients develop serious complications, such as acute respiratory distress syndrome (ARDS) and multiple organ failure. Knowledge has accumulated on the basic mechanisms of the pathogenesis of COVID-19; it is based on immune dysregulation in the excessive response to viral infection [1] and the development of cytokine storms [2,3] or hyperinflammatory COVID-19-related syndromes [4].

The uncontrolled immune response and hyperinflammation of COVID-19 were the reasons for prescribing widely used drugs for autoimmune rheumatic disease (AIRD) treatment. The phenomenon of drug reuse refers to the use of antirheumatic drugs for the treatment of COVID-19 [5]. Many years of worldwide experience have been accumulated in the use of ADs, colchicine, calcineurin inhibitors (cyclosporine A and tacrolimus), glucocorticoids, TNF-alpha inhibitors (infliximab), interleukin (IL)-6 inhibitors (tocilizumab, sarilumab, and siltuximab), IL-1 inhibitors (anakinra, canakinumab), and Janus kinase (baricitinib and tofacitinib) and other drugs. 

Medications from the antimalarial series, whose anti-inflammatory and immunomodulatory properties are the reasons for their use in coronavirus infections, are very interesting. Hydroxychloroquine (HCQ) was one of the first drugs to be investigated for the treatment of COVID-19 [5]. Although many clinical trials have been published since the first wave of the pandemic all over the world, the presented results on its efficacy and safety in COVID-19 aspects are highly discordant. Some contradictory factors of AD clinical applications still need to be comprehensively analyzed. For example, the reason why antimalarial drugs have not been able to repeat their success compared to the field of rheumatology in the treatment of COVID-19. With regard to COVID-19 pharmacotherapy, the primary focus has been placed on the antiviral properties of these drugs in high doses [4,6]. On the contrary, in rheumatology, the use of low-dose ADs is based on their anti-inflammatory, immunomodulatory, and anticoagulant effects. This article summarizes the current knowledge about the mechanisms of action of ADs and shows the distinctions between their application, recommendations, and side effects in two groups of patients: those with rheumatic diseases and COVID-19.

## 2. Pathogenetic Stages of SARS-CoV-2

**Figure 1:**
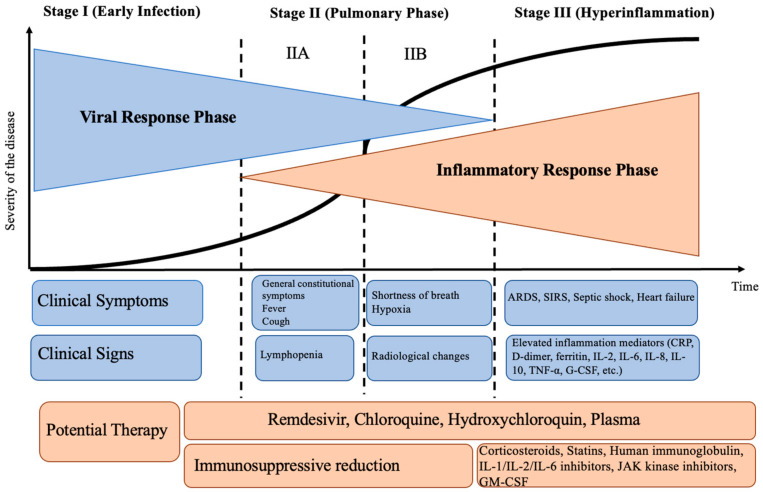
Stages of the progression of SARS-CoV-2. Notes: ARDS: acute respiratory distress syndrome; SIRS: systemic inflammatory response syndrome; CRP: C-reactive protein; IL: interleukin; TNF-α: tumor necrosis factor α; GM-CSF: granulocyte-macrophage colony-stimulating factor.

Currently, for classification purposes, a conditional division of infection into stages is used, reflecting clinical symptoms, outcomes, and potential responses to therapy (Figure 1). The early phase begins when the virus replicates with local spread and a limited immune response. Clinical symptoms are non-specific: fever, malaise, sore throat, dry cough, dysgeusia, and dyssomnia, or no symptoms at all [6]. In the second instance, the virus migrates to the lower respiratory tract, infects cells of the pulmonary alveolar epithelium, and leads to the development of pneumonia and hypoxia. The third phase is characterized by the development of hyperinflammatory syndrome, hypercoagulation, and “thrombus inflammation” [7]. Figure 1 [8] illustrates the stages of the escalation of COVID-19 infection with characteristic symptoms and potential therapies. 

## 3. Antimalarial Drugs in Rheumatology

The use of ADs in rheumatology dates back to the 19th century, when quinine obtained from cinchona bark was first used in the treatment of patients with the discoid form of systemic lupus erythematosus (SLE) [9]. During World War II, quinacrine was used in Pacific Island populations as a prophylaxis and treatment for malaria [10]. At the same time, a positive effect of the drug was observed on the course of rheumatoid arthritis (RA). Less toxic AD derivatives (chloroquine phosphate (Delagil) and hydroxychloroquine sulfate (Plaquenil)) were synthesized in the 40s of the last century; they have a wide range of therapeutic effects and similar molecular mechanisms of pharmacological activity [11]. The chemical difference is the replacement of the ethyl group with a hydroxyethyl group for HCQ [12]. There is evidence of a lower degree of toxicity and fewer drug interactions with HCQ [12].

In therapeutic doses, ADs have photoprotective, anti-inflammatory, immunomodulatory, antioxidant, antiplatelet, hypolipidemic, hypoglycemic, and analgesic effects. Their pleiotropic effect demonstrates a wide range of effects on the hemostatic system, lipid, and glycemic profiles, which leads to a reduction in the risk of cardiovascular events [13]. The positive effect of HCQ has been shown predominantly in patients with SLE with skin lesions, arthritis, and serositis [14], as well as a thromboprotective effect in antiphospholipid syndrome (APS) [15]. Long-term use in these patients significantly reduces the incidence of thromboembolic complications [16]. Currently, ADs are prescribed more frequently as a basic therapy for RA and SLE of low and moderate activity, in particular, in patients with SLE with skin, joint, constitutional syndromes, and serositis [17]. 

The prescription of HCQ to manage SLE during pregnancy has been adopted as the standard of care in recent years, with multiple national and international rheumatology guideline groups recommending it for all women with lupus [18,19,20,21]. Previous data did not show an increase in congenital anomalies in infants exposed to in utero HCQ for malaria, and more recent studies have confirmed this finding [22,23]. The data of the meta-analysis show that taking HCQ throughout pregnancy decreased the odds of high SLE activity and did not significantly affect pregnancy outcomes [24,25]. Furthermore, HCQ administration during pregnancy had no impact on fetal loss, preterm delivery, or preeclampsia and was associated with a lower probability of preterm delivery; this benefit was not found in women with highly active SLE. Specifically, these studies noted that prematurity, intrauterine growth restriction, congenital malformations, low birth weight, and stillbirth were not associated with HCQ. Interestingly, the retrospective study in the Johns Hopkins cohort involving 304 patients and 398 pregnancies showed that there was a higher flare rate defined by physician-assessed activity, with a hazard ratio (HR) of 1.59 (95% CI: 1.27 to 1.96). This effect was modified by the use of HCQ, with an estimated HR for flares during pregnancy of 1.83 (95% CI 1.34 to 2.45) in patients without HCQ and 1.26 (95% CI 0.88 to 1.69) in patients with HCQ [26]. This study concluded that there was an increase in the incidence of flares during pregnancy and 3 months postpartum but that the use of HCQ appeared to reduce the risk of flares during and after pregnancy. Another meta-analysis of published observational studies of HCQ suggested a decrease in preeclampsia and gestational hypertension [27].

Recent retrospective and prospective studies suggested a benefit of HCQ in lowering the risk of cardiac manifestations of neonatal lupus (cardiac NL) and significantly decreasing the incidence of congenital heart blocks (CHBs) in the pregnancies of patients positive for antiSSA/Ro with systemic lupus erythematosus [28,29,30]. The results were suggested by a multinational historical cohort study, where HCQ was associated with a reduction in the CHB recurrence rate by 60% [31]. The next open-label multicenter trial [32] supported that HCQ significantly reduces CHB recurrence below the historical rate by >50%, suggesting that this drug should be prescribed for the secondary prevention of fetal cardiac disease in antiSSA/Ro-exposed pregnancies. 

The mechanism of action and clinical effects of ADs in AIRD are presented in more detail in Table 1.

## 4. Mechanism of Action of the Aminoquinoline Series of Drugs against SARS-CoV-2

Early studies have shown the effectiveness of ADs against various viruses containing RNA (hepatitis A and C, rabivirus, and Ebola fever) and viruses containing DNA (hepatitis B and the herpes simplex virus) [47,48]. In vitro and in vivo studies have reported the effectiveness of ADs in treating coronavirus infections, including human virus subtypes CoV-229E [49], HCoV-OC43 [50], and SARS-CoV-1 [51].

The antiviral effect of ADs is associated with the inhibition of the process of viral replication by binding viral particles to cell surface receptors [48]. It is known that coronavirus infection occurs through the endosomal route [52]. By increasing the pH level of intracellular endosomes and lysosomes [48], ADs prevent the fusion of the virus with the cell by disrupting vital cellular pathways and subsequent replication [53]. An elevation in endosomal pH induced by ADs inhibits cathepsin formation, which necessitates an acidic environment for the optimal cleavage of the SARS-CoV-2 spike protein [54]. Consequently, by modulating endosome acidification, virus/cell fusion with host cells and subsequent viral replication is prevented [55]. In vitro studies have demonstrated that ADs inhibit SARS-CoV-2 replication at low micromolar concentrations [56]. Recent research also indicates that HCQ impedes the interaction between the spike protein and the cell membrane by binding to gangliosides, thereby preventing viral entry into the cell [53]. Furthermore, the inhibitory effect of these drugs on SARS-CoV-2 is attributed to their influence on both the entry and intracellular stages of viral replication in Vero E6 cells [54]. It is known that many coronaviruses, including SARS-CoV-2, can enter the upper respiratory tract through sialic acid receptors, a process essential for viral replication [57]. The inhibitory ability of predominantly HCQ towards sialic acid [50] was previously known. SARS-CoV-2 is thought to enter through binding to sialic acid receptors via the α2–6 linkage, which is most pronounced in the epithelium of the conjunctiva, cornea, and nasolacrimal regions [58]. Consequently, the accidental entry of viral particles into the eyes or nasolacrimal ducts can lead to successful penetration into the host body.

Another mechanism of action of aminoquinoline drugs is associated with a similar “point of application” for SARS-CoV-2. These are the surface receptors of the angiotensin-2 converting enzyme (ACE2). They are located on the surface of the lungs, heart, kidneys, and intestines [59]. By influencing the final glycosylation process of the receptors, drugs from this group prevent the binding of SARS-CoV-2 to the ACE2 receptors expressed on pneumocytes and further replication [59].

ADs have anti-inflammatory and immunomodulatory effects in the immunopathogenesis of both AIRD and Coronavirus. They are caused by the activation of the immune system, the inhibition of antigen processing, and self-antigen presentation mediated by the major histocompatibility complex (MHC) in antigen-presenting cells (APCs). A decrease in the level of activated T-cells causes a decrease in the production of cytokines generated by T-cells and B-cells (IL-1, IL-6, TNF, and IFN-gamma) [60,61]. By changing cellular pH, ADs affect the functioning of Toll-like receptors (TLRs) [36].

There is evidence for the effect of ADs on the MAP kinase cascade [62]. By inhibiting multifunctional intracellular signaling pathways containing mitogen-activated protein kinases, ADs indirectly control metabolism, proliferation, cell motility, assembly, and propagation of the virion. ADs reduce the production of pro-inflammatory mediators by activating antiCD8+ T-cells and inhibiting the production of IL-1, IL-6, and TNF-α [60]. In the initial stage, SARS-CoV-2 has the ability to increase the secretion of Th-2 helper lymphocytes and anti-inflammatory cytokines IL-4 and IL-10 [63]. 

**Figure 2:**
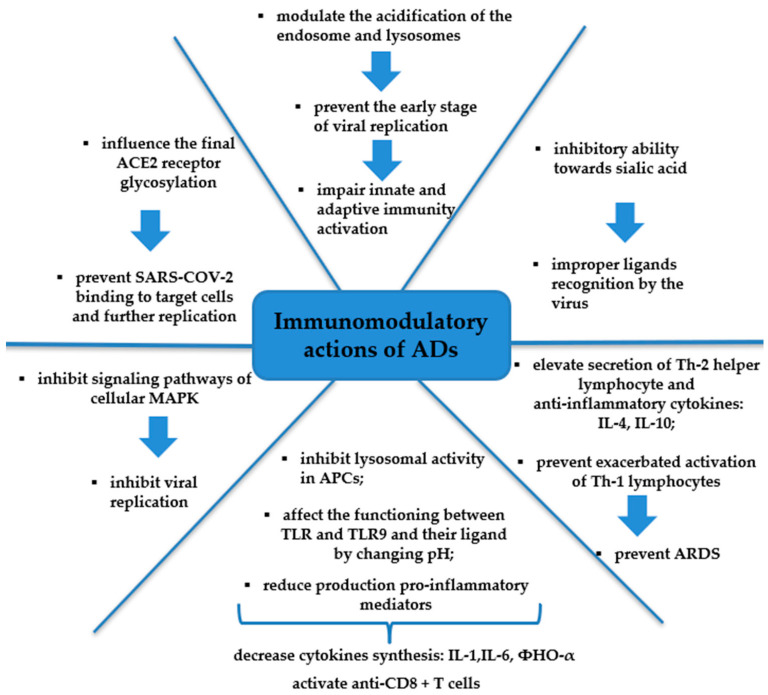
The mechanisms of antiviral and immunomodulatory activity ADs in SARS-CoV-2 infection. Notes: ADs: antimalarial drugs, ACE2: angiotensin-converting enzyme 2; IL: interleukin; APCs: antigen-presenting cells; TLRs: Toll-like receptors; MAPK: mitogen-activated protein kinase; ARDS: acute respiratory distress syndrome.

ADs inhibit the excessive activation of Th-1 lymphocytes, which play a key role in the development of ARDS [48]. Beyond preventing viral entry, CHQ inhibits the secretion of IL-2, which is involved in the differentiation of T-2 cells into the Th-2 subset [64]. Thus, the proliferation of Th-2 lymphocytes is diminished. Therefore, it is proposed that CQ and HCQ impair the immune response to SARS-CoV-2 infection and prevent the progressive course of the disease. In summary, AMDs in coronavirus infection interfere with ACE2 to prevent viral invasion, increase the endosomal pH required for the fusion of the virus, and show mild immune suppression. Presumably, decreasing the excess production of pro-inflammatory markers can deteriorate the severity of the viral infection. Figure 2 demonstrates the possible model of AD antiviral and immunomodulatory activity in SARS-CoV-2 infection.

## 5. Experience in the Treatment of SARS-CoV-2

The excellent safety profile and proven antiviral activity served as a justification for the use of 4-aminoquinoline drugs in the treatment of SARS-CoV-2. However, since the onset of the pandemic, there has been very limited evidence of positive results in practical applications. 

### 5.1. Research Contributing to the Successful HCQ Application in COVID-19 Treatment

There is evidence of a positive effect of monotherapy or a combination of ADs with azithromycin on the course of SARS-CoV-2, resulting in decreased virological load and improvements in clinical and radiological parameters. Fontana et al. [65] observed a significant reduction in seroconversion rates in patients treated with HCQ and azithromycin. At the same time, 83% and 93% of patients on the seventh and eighth days of observation, respectively, provided a negative result in a nasopharyngeal smear test for SARS-CoV using the PCR method. Million et al. [63] reported favorable clinical outcomes and a virological cure in 91.7% of patients (*n* = 1061) treated with a combination of HCQ and azithromycin. The duration of treatment was 10 and 5 days, respectively. In a comparative study, Gautret et al. [66] also reported a decrease in the rate of seroconversion in the HCQ treatment group when combined with azithromycin (*p* = 0.001). Virological cure by the sixth day of observation was achieved by 100% of patients on the combination therapy, with 57.1% and 12.5% in the HCQ treatment group and control group, respectively (*p* < 0.001).

Studies of the first wave of the pandemic showed a reduction in the risk of COVID-19-associated mortality in patients treated with HCQ monotherapy (RR 0.44; 95% CI 0.29–0.67) [67]. According to another multicenter study, HCQ provided a reduction in the death risk ratio by 66% (95% CI 11.6–15.5%), and in combination with azithromycin by 71% (95%; CI 17.3–23.0%) compared to any treatment (*p* < 0.001) [68].

### 5.2. Research Contributing to the Failure of HCQ Application in COVID-19 Treatment

On the contrary, open-label RCTs [69,70] did not show any improvement in clinical outcomes for patients with varying degrees of COVID-19 infection when treated with azithromycin combinations. According to a double-blind, placebo-controlled study [70], HCQ treatment did not affect the severity of infection between the study and control groups. Furthermore, side effects were 21% higher (92 of 212 patients) compared to the placebo group (46 of 211 patients, *p* < 0.001).

Subsequent large-scale studies conducted in the USA [71,72] and UK [73] also demonstrated negative results from the use of ADs. For example, a large observational study in New York [72] assessed the association between HCQ use and intubation or death (*n* = 1376). It was revealed that patients who initially received HCQ were more severely ill; moreover, HCQ use was not associated with a significant reduction in the risk of intubation or death (RR 1.04; 95% CI 0.82–1.32).

The RECOVERY study [73] (*n* = 4716) compared groups of patients receiving HCQ (*n* = 1561) and standard therapy (*n* = 3155). It was found that the 28-day mortality rate in the HCQ treatment group was no lower than in the control group (26.8% vs. 25%; RR 1.09; 95% CI 0.96–1.23, *p* = 0.18). Moreover, in the subgroup of patients not requiring mechanical ventilation, the likelihood of subsequent intubation or death was higher in the HCQ group (29.8% vs. 26.5%, RR 1.12; 95% CI 1.01–1.25). Similar results were obtained in the SOLIDARITY study [74]: the mortality rate of hospitalized patients was 104 of 947 patients in the HCQ group and 84 of 906 patients in the control group (11.0% vs. 9.3%; RR 1.19; 95% CI 0.89–1.59; *p* = 0.23). Thus, HCQ has a negligible effect on mortality, mechanical ventilation requirements, and length of hospital stay. Table 2 demonstrates the results of large, well-conducted clinical studies on HCQ in COVID-19 treatment.

In December 2020, the World Health Organization (WHO) announced a strong recommendation against the use of ADs in the treatment of COVID-19, regardless of the severity of the disease and the duration of symptoms [78]; in March 2021, recommendations against the use of HCQ for preventive purposes were published [79].

**Figure 3:**
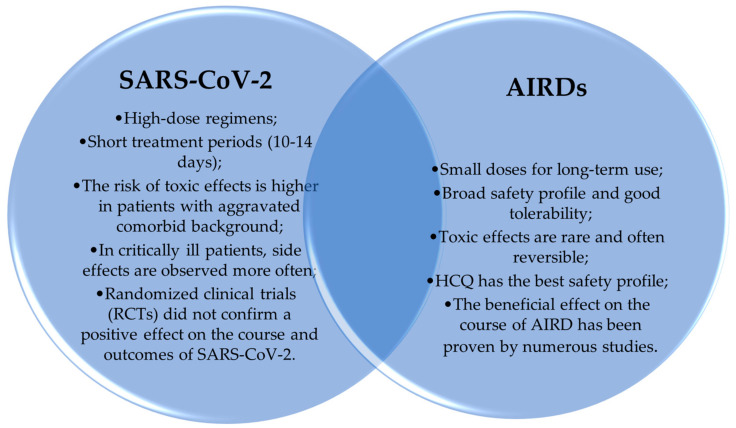
Comparison of AD use in the treatment of rheumatic diseases and coronavirus infection. Notes: AIRDs: autoimmune rheumatic diseases; RCTs: randomized clinical trials; HCQ: hydroxychloroquine.

Unfortunately, in the treatment of coronavirus infections, antimalarial drugs have not been able to replicate their success in the field of rheumatology. Possibly, in addition to the different points of application of ADs in the context of these pathologies, this failure is explained by a different course of the infection disease (mild vs. severe), the high dosage in a short-treatment period (400–1200 mg), and COVID-19’s frequent association with other chronic diseases (mainly cardiovascular, renal, and respiratory disorders) [5,57,80]. Compared to SARS-CoV-2, the dose of ADs administered to AIRD is significantly lower, and the time required to reach drug saturation is even longer [11,14,18]. The complete therapeutic efficacy may become apparent at a later time, and the probability of serious adverse effects is minimal. In particular, antimalarial myopathy, cardiomyopathy, or maculopathy have not been frequently recorded [10,18]. Figure 3 demonstrates the fundamental differences in the therapeutic approaches and related consequences to AD administration in AIRDs and COVID-19. 

## 6. Side Effects of ADs

The need to prescribe high doses of ADs for SARS-CoV-2 necessitates the strict monitoring of side effects, especially in patients with critical indicators of renal and hepatic function [80].

The most serious side effects of ADs include cardiotoxic and pro-arrhythmogenic effects due to the prolonged QT interval [80,81]. The WHO pharmacovigilance database recorded 83 cases of “torsades de pointes” and other types of ventricular tachycardia associated with HCQ use, seven of which were fatal [77]. In a systematic review, Karia et al. [80] (*n* = 1350) studied the side effects of patients with confirmed COVID-19 while taking HCQ. The most common side effect was QT prolongation (2.7%). It is worth noting that the therapy included other drugs that prolong the QT interval, such as azithromycin and/or levofloxacin. The least common side effects were diarrhea (1.25%), nausea/vomiting (1.18%), and acute kidney injury (0.37%). The US Center for Disease Control and Prevention (CDC) has suggested that due to the long half-life of HCQ (more than 40 days), patients may be at risk of adverse cardiac events and drug interactions even after the completion of treatment [66].

Another serious side effect is the retinal toxicity of ADs [12,82]. HCQ has the highest level of toxicity [11]. With long-term use, damage to the lysosomal structures of the photoreceptors of the retinal epithelium leads to retinopathy [82], which can progress to blindness due to retinal destruction. Reversible changes in the cornea and loss of visual field are also possible. The need for regular ophthalmological examination when taking antimalarial drugs is emphasized.

Hyperpigmentation of the skin, cartilage of the ears and nose, trachea, and joint tissues has been reported in patients regularly receiving HCQ [83]. Rare cutaneous side effects include acute generalized exanthematous pustulosis, multiform erythema, and toxic epidermal necrolysis [84]. The observed skin rashes may be associated with an imbalance of the immune system in patients treated with ADs [84]. The most common side effects are dyspepsia, nausea, vomiting, itching, headaches, and vestibular disorders [80,85,86]. Cases of agranulocytosis and hemolysis have been reported in patients with glucose-6-phosphate deficiency [11].

## 7. Conclusions

For almost four years, the COVID-19 pandemic has posed a serious threat and has been considered a global health emergency. The fact that the infection is growing rapidly has led to the search for quick and effective treatment methods to prevent its further spread. ADs act as a “candidate” for the treatment of a new coronavirus infection due to previously studied mechanisms of antiviral activity, such as interactions with endocytic pathways, the prevention of the glycosylation of ACE2 receptors, blocking sialic acid receptors, and reducing the manifestations of cytokine storms. Despite numerous side effects, they have been widely used in clinical trials around the world. The results of studies in some countries showed promising results, but large randomized clinical trials (RCTs) refuted the fact that there was a positive effect on the course and results of COVID-19. Furthermore, the need for high doses in the treatment of viral infection increases the likelihood of gastrointestinal side effects, prolongation of QT, and retinopathy. These circumstances do not allow their use for the treatment and prevention of COVID-19.

## Figures and Tables

**Table 1:** Use of ADs for AIRDs.

Disease	Priority of Therapy	Mechanism of Action	Clinical Effects
Systemic lupus erythematosus	It is a first-line therapy drugs of choice for damage to the skin, joints, and constitutional disorders. There is evidence of the advisability of including ADs in combination therapies in patients with nephritis, vasculitis, and central nervous system damage.	Stabilizes cell membrane organelles and lysosomal enzymes; increases the endosomal pH => decreases interferon production => inhibits the stimulation of autoreactive B lymphocytes; inhibits antigen-presenting cells; inhibits mRNA expression => reduces the synthesis of pro-inflammatory mediators (IL-1 β, IL-6, and TNF-α); inhibits lipid oxidation processes [33].	Reduces disease activity [33,34] and the risk of multiorgan damage and musculoskeletal and skin syndromes [33]; Reduces the frequency of serositis and prevents the exacerbations of SLE [34]; reduces fatigue and general weakness, reduces disease activity and infection risk [35]; increases long-term survival rates [18,19,20]; prevents multiorgan damage, photoprotective, thromboprotective, osteoprotective [33,34], nephroprotective [36]; preventive effect of neuro-lupus [37]; antithrombotic [34,38], cardioprotective due to hypoglycemic and hypolipidemic properties [13]; steroid-sparing effect [35]; reducing the risk of complications during pregnancy [36]. It can be used as part of a combination therapy if methotrexate is not sufficiently effective [38].
Rheumatoid arthritis	It is a second-line therapy used as part of a combination or monotherapy for RA. Can be used in the case of intolerance to other basic anti-inflammatory drugs in the early stage of low activity and absence of an unfavorable prognosis.	Antagonism of Toll-like receptors (TLR) => suppression of the immune response [36]; interference with the processes of antigen presentation and lysosomal oxidation [36]; inhibition of the production of RF antibodies, proteases, and collagenases => preventing the destruction of cartilage, inhibition of phospholipase A2 [11]; decreased synthesis of pro-inflammatory cytokines (IL-1β, IL-6, and TNF-α) [11].	
Sjögren disease (SD)	It is a first-line therapy in combination with muscular-articular syndrome [39] recommended for patients with SD with significant fatigue and systemic symptoms for 6–12 months [40].	Reduction of the factors that activate B cells in tear fluid [41,42].	Reduces arthralgia, myalgia, and fatigue syndromes [40]; reduces disease activity, increases salivation, reduces dry eye syndrome’s impact on the integrity of the cornea [41,42]; Reduces cardiovascular risk due to the effect of lipidemia and glycemia [13].
Sarcoidosis	It is a second-line therapy recommended for the cutaneous form of sarcoidosis in the case of the ineffectiveness of glucocorticoid local application [43].	Decreased secretion of pro-inflammatory cytokines [44]; inhibition of the function of antigen-presenting cells => reduction in antigen processing and presentation through the major histocompatibility complex (MHC) system => reduction in granulomatous damage by T lymphocytes [45].	Prevents sarcoid lesions in the skin in monotherapy or in combination with glucocorticoids [44]. Favorable effect on hypercalcemia and hypercalcemia in sarcoidosis (in combination with glucocorticoids) [45]. Promotes the regression of lung damage in the pulmonary form [45].
Dermatomyositis	It is allowed to be prescribed with a minimal degree of activity to reduce the manifestations of skin syndromes in combination with glucocorticoids.	Inhibition of phospholipase A2; decreased synthesis of pro-inflammatory cytokines; decreased phagocytic and chemotactic activity of immune cells; inhibition of the formation of immune complexes; antioxidant effects [46].	Reduces skin manifestations, including the juvenile form of dermatomyositis [46].

**Table 2:** Overview of research on HCQ in COVID-19 treatment.

Authors	Study Design	Quantity	Results
Gautret et al. [66]	Open-label and non-randomized	80	Viral load reduction/disappearance with HCQ in combination with azithromycin
Yu et al. [75]	Retrospective cohort	568	Significant association with decreased mortality in critically ill COVID-19 patients and attenuation of inflammatory cytokine IL-6 level.
Million et al. [63]	Retrospective analysis of a case series	1061	Improved clinical outcomes, mortality rates, and virological cure
Arshad et al. [68]	Multicenter retrospective observational study	2541	Association with reduced COVID-19-associated mortality in HCQ monotherapy or in combination with azithromycin
Furtado et al. [69]	Open-label and randomized	447	HCQ with azithromycin did not improve clinical outcomes
Skipper et al. [76]	Randomized, double-blind, and placebo-controlled	491	HCQ did not substantially reduce symptom severity in outpatients with early, mild COVID-19
Pan et al. [74]	Randomized multicenter retrospective	954	Minimal or no effect of HCQ on hospitalized patients, as indicated by the overall mortality, initiation of ventilation, and duration of hospital stay
Rosenberg et al. [72]	Retrospective multicenter cohort study	1438	Monotherapy with HCQ or in combination with azithromycin was not associated with differences in in-hospital mortality
Jankelson et al. [77]	Systematic review	1515	QT prolongation was registered in approximately 10% of COVID-19 patients treated with HCQ, and there were two cases of ventricular arrhythmia
Horby et al. [73]	Randomized, controlled, open-label platform	1561	HCQ did not cause a lower incidence of death at 28 days
